# A unique association of bifacial weakness, paresthesia and vestibulocochlear neuritis as post-COVID-19 manifestation in pregnant women: a case report

**DOI:** 10.11604/pamj.2021.38.30.27646

**Published:** 2021-01-13

**Authors:** Jehanne Aasfara, Amal Hajjij, Hatim Bensouda, Hamid Ouhabi, Fouad Benariba

**Affiliations:** 1 Department of Neurology, Cheikh Khalifa International University Hospital, Faculty of Medicine, Mohammed VI University of Health Sciences (UM6SS), Casablanca, Morocco,; 2 Department of Otolaryngology, Head and Neck Surgery, Cheikh Khalifa International University Hospital, Faculty of Medicine, Mohammed VI University of Health Sciences (UM6SS), Casablanca, Morocco,; 3 Department of Otolaryngology, Head and Neck Surgery, Mohammed V Military Training Hospital, Rabat, Morocco

**Keywords:** Bifacial weakness and paresthesia, vestibulocochlear neuritis, Guillain Barré syndrome, SARS-CoV-2, case report

## Abstract

SARS-CoV-2 is an infection due to a novel virus belonging to the coronavirus family. Since December 2019, first human cases of COVID-19 have been identified in Wuhan (China) and rapidly has been progressed to a global pandemic declared by the world health organization (WHO) on March 11^th^ 2020. The major complication of COVID-19, is pneumonia, but other presentations like cardiovascular and neurological complications have been reported. Herein, we report a first case of pregnant women presented with bifacial weakness and paraesthesia (BFP) associated to a vestibulocochlear neuritis as post-COVID-19 manifestation. This is a 36-year-old Moroccan female patient with a history of SARS-CoV-2 positive 6 weeks before admission. She presented to the emergency department with rapid bifacial paralysis, bilateral lower extremity paresthesia, vertigo, nausea, vomiting and right auricular pain. An acute stroke was ruled out after neurological examination and brain MRI. Clinical presentation, neurophysiological, audiometry and videonystagmography workup additionally to CSF findings were suggestive of a variant of Guillain Barré Syndrome (GBS), which is BFP associated to right vestibulocochlear neuritis. The patient was treated with Intravenous immunoglobulins (IVIG) therapy associated with intravenous steroids. The patient made a complete recovery of the right facial palsy and the sensorineural hearing loss but still have tingling in lower limbs and left facial palsy at 2 weeks´ follow-up. BFP can be induced by COVID-19 as a postinfectious immune-mediated complication. Regarding the pathophysiology of vestibular neuritis, is probably similar to other viral infection causing nerve damage. Clinicians should consider the association of vestibulocochlear neuritis and BFP as a post SARS-CoV-2 manifestation.

## Introduction

The outbreak of Coronavirus Disease 2019 (COVID-19) which started in December 2019, in China, has rapidly spread around the world and has become a pandemic. Although neurological manifestations appear fairly rare, they can lead to major complications. Clinicians should be aware of the variety of neurological presentation to avoid misdiagnosis or delayed diagnosis [1]. Herein, we report a first case of pregnant women presented with bifacial weakness and paraesthesia (BFP) associated to a vestibulocochlear neuritis as post-COVID-19 manifestations.

## Patient and observation

A 36-year-old pregnant women at 37 weeks of gestation with a history of SARS-CoV-2 positive 6 weeks before, was admitted to the emergency room, for a sudden onset of vertigo, nausea, vomiting one day before admission complicated by left-sided facial weakness and fullness of the right ear with tinnitus. She denied a previous history of vertigo, head trauma, otitis or tick bite. On admission, a normal pregnancy was confirmed by an obstetrical examination and ultrasound. Vital signs including blood pressure were normal. Neurological and vestibular examination showed a reduced tendon reflexes in lower limbs with preserved strength, a spontaneous horizontal and rotatory left-beating nystagmus grade 3 associated to a left peripheral facial palsy grade IV of Brackman and House. Otological examination showed a normal tympanic membrane bilaterally with no vesicles on external auditory canal. After 24 hours, she presented a right peripheral facial palsy and asymmetric distal numbness in the lower limbs and left fingers.

**Figure 1:**
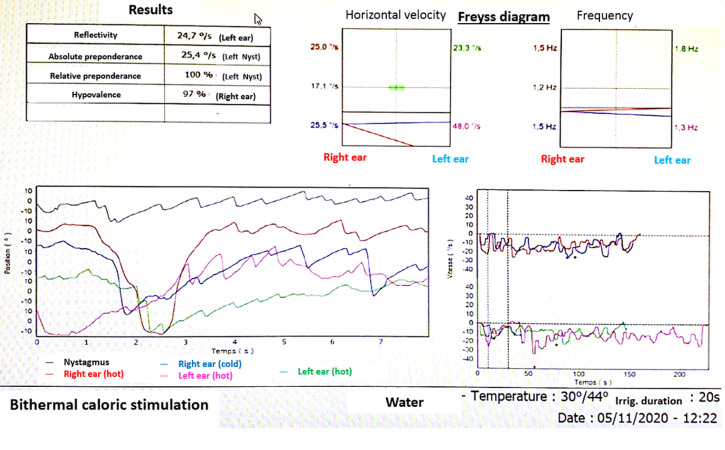
videonystagmography showing right vestibular areflexia on caloric examination

Oto-neurological tests revealed, severe right sensorineural hearing loss on pure tone audiometry (Hearing level at 80 dB on 250 Hz, 75 dB on 500 Hz, 70 dB on 1000 Hz, 70 dB on 2000 Hz, 80 dB on 4000 Hz and 80 dB on 8000 Hz). Videonystagmography showed complete right vestibular areflexia on caloric examination with left-beating spontaneous horizontal and torsional nystagmus without extrinsic ocular motricity deficit neither a gaze nystamus (Figure 1). Brain and spinal cord MRI explorations were normal. Electromyography and nerve conduction studies showed isolated absence of F waves in right tibial and peroneal nerves supporting the diagnosis of demyelinating pattern of Guillain Barré Syndrome (GBS). Diagnostic workup including complete blood count, fasting glucose, erythrocyte sedimentation rate, serum angiotensin-converting enzyme level, antinuclear antibody, anti-DNA, ANCA and anti-ganglioside were negative. The lumbar puncture showed an albuminocytological dissociation (raised protein levels (0.8g/dL; normal range <0.4 with normal cell counts and glucose). Cerebrospinal fluid (CSF) polymerase chain reaction assay (PCR) for several viruses, including, SARS-CoV-2, Cryptococcus, Mycobacterium tuberculosis, Lisetria, Escherichia coli, was negative, neither was serology of Campylobacter jejuni, Epstein - Barr virus, Cytomegalovirus, TPHA-VDRL and Borrelia. Blood serology revealed SARS-CoV-2 IgM negative and IgG positive antibody. Testing by RT-PCR was negative.

**Figure 2:**
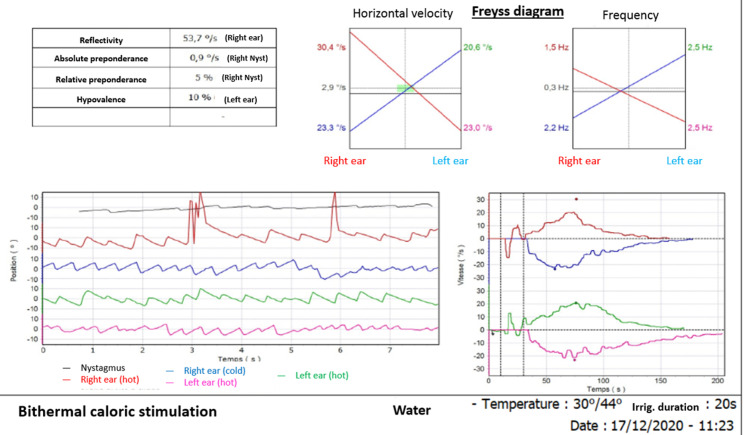
caloric examination at 6 weeks follow-up showing a complete recovery of the right vestibular loss on low frequencies with a remaining slight spontaneous left-beating nystagmus

Clinical presentation, neurophysiological, audiometry and videonystagmography workup additionally to CSF findings were suggestive of BFP and right vestibulocochlear neuritis. Intravenous immunoglobulins (IVIG) therapy was started at a dose of 0.4 g/kg for 5 days associated to intravenous steroids (1mg/Kg) for 10 days. The patient made a complete recovery of the right facial palsy and the sensorineural hearing loss but still had tingling in lower limbs and left facial palsy at 2 weeks´ follow-up. Nerve conduction studies were repeated a week after and showed rapid restoration of F waves in initially involved nerves. Vertigo improved over time with vestibular rehabilitation exercises. Videonystagmography performed 6 weeks after onset, showed a total recovery of vestibular loss on caloric examination on the right ear with remaining slight spontaneous left-beating nystagmus (Figure 2). Otherwise, the rest of the pregnancy progressed normally and she had an uncomplicated spontaneous vaginal delivery of a healthy female baby at 40 weeks´ gestation.

## Discussion

Herein, we report a patient with a 6 weeks´ history of SARS-CoV-19 infection who developed rapidly progressive bilateral facial palsy, extremity paresthesia, and right vestibulocochlear neuritis. Our clinical and electrophysiological findings were consistent with bifacial weakness and paresthesia subtype of GBS. In addition, our case highlights the possibility of occurrence of BFP and a vestibulocochlear neuritis as a post infectious manifestation of COVID-19. BFP is a variant of GBS which is an acute immune-mediated neuropathy. Because of molecular mimicry, antibodies produced after an infection cross react with neuronal antigens causing axonal and/or demyelinating neuropathy [2]. The underlying mechanisms of GBS in COVID-19 are not well understood. It can be likely a postinfectious immune-mediated mechanism or infrequently a para-infectious phenomenon [3]. Hutchins *et al*. reported a case of BFP in a patient with recent diagnosis of COVID-19 and positive HSV serologies which make the cause and effect relationship between SARS-CoV-2 and BFP, difficult to establish [4]. In our case, only SARS-CoV-2 serology was positive.

Abu Rumeileh *et al*. reported in a systematic review of 73 cases, that classic GBS was the most form described, with sensorimotor presentation and acute inflammatory demyelinating polyneuropathy. Rarely, variants like Miller Fisher syndrome or BFP were reported. CSF analysis showed mostly (71%) an albuminocytological dissociation but CSF SARS-CoV-2 RNA was constantly absent in the 73 cases [5]. In our case, albuminocytological dissociation was found with negative CSF SARS-CoV-2 RNA. Nerve conduction studies showed isolated absence of F waves in 2 nerves (tibial and peroneal). According to Wakerley *et al*. (2015) diagnostic criteria of BFP, our case accomplishes bilateral facial weakness, distal paresthesia, limb areflexia, without ophthalmoplegia, ataxia, limb or neck weakness [2]. Based on paraclinical findings, we hypothesize that BPF is a post infectious complication of SARS-CoV-2 infection. Moreover, our patient presented unilateral sensorineural hearing loss with vestibular areflexia miming either labyrinthitis or a retrocochlear hearing loss. Indeed, no suspicious lesion or enhancement of the labyrinth were found on contrast enhanced MRI of internal auditory canal and cerebellopontine angle. Malayala *et al*. reported a case of acute vestibular neuritis secondary to SARS-CoV-2 infection witch the pathophysiology is probably analogous to other viral infection causing vestibular nerve damage [6,7]. Moreover, our patient rapidly improves after IGIV and corticosteroid therapy suggesting an inflammatory reaction rather than direct neuronal damage [8]. To the best of our knowledge, this is the first case of an association of Vestibulocochlear neuritis and BFP in post SARS-CoV-2 infection.

## Conclusion

This case report, highlights the possibility of association between a variant of Guillain-Barré syndrome (BFP) and vestibulocochlear neuritis and SARS-CoV-2 infection. Post-infectious immunological mechanisms are thought to be the cause of this manifestations. The exact pathophysiology remains unclear and need further studies to clarify these conditions.
